# The four-item PRECISE-DAPT score identifies coronary artery bypass grafting patients with increased risk for post-discharge major bleeding

**DOI:** 10.1093/ehjcvp/pvae060

**Published:** 2024-08-20

**Authors:** Philip Enström, Andreas Martinsson, Mary Rezk, Susanne Nielsen, Erik Björklund, Maya Landenhed-Smith, Emily Pan, Anders Jeppsson

**Affiliations:** Department of Cardiothoracic Surgery, Sahlgrenska University Hospital, Gothenburg 41345, Sweden; Department of Molecular and Clinical Medicine, Institute of Medicine, Sahlgrenska Academy, University of Gothenburg, Gothenburg 41124, Sweden; Department of Molecular and Clinical Medicine, Institute of Medicine, Sahlgrenska Academy, University of Gothenburg, Gothenburg 41124, Sweden; Department of Cardiology, Sahlgrenska University Hospital, Gothenburg 41345, Sweden; Department of Cardiothoracic Surgery, Sahlgrenska University Hospital, Gothenburg 41345, Sweden; Department of Molecular and Clinical Medicine, Institute of Medicine, Sahlgrenska Academy, University of Gothenburg, Gothenburg 41124, Sweden; Department of Cardiothoracic Surgery, Sahlgrenska University Hospital, Gothenburg 41345, Sweden; Department of Molecular and Clinical Medicine, Institute of Medicine, Sahlgrenska Academy, University of Gothenburg, Gothenburg 41124, Sweden; Department of Molecular and Clinical Medicine, Institute of Medicine, Sahlgrenska Academy, University of Gothenburg, Gothenburg 41124, Sweden; Department of Medicine, Southern Älvborg Hospital, Borås 50182, Sweden; Department of Cardiothoracic Surgery, Sahlgrenska University Hospital, Gothenburg 41345, Sweden; Department of Molecular and Clinical Medicine, Institute of Medicine, Sahlgrenska Academy, University of Gothenburg, Gothenburg 41124, Sweden; Department of Surgery, Central Finland Hospital Nova, Jyväskylä 40630, Finland; Department of Cardiothoracic Surgery, Sahlgrenska University Hospital, Gothenburg 41345, Sweden; Department of Molecular and Clinical Medicine, Institute of Medicine, Sahlgrenska Academy, University of Gothenburg, Gothenburg 41124, Sweden

**Keywords:** Coronary artery bypass grafting, Antithrombotic treatment, Dual anti platelet therapy, Bleeding complications, PRECISE-DAPT score

## Abstract

**Aims:**

Early identification of patients with increased bleeding risk increases the possibility to individualize antithrombotic treatment. We validated the PRECISE-DAPT score, originally developed to estimate bleeding risk in patients on dual antiplatelet therapy (DAPT) after percutaneous coronary intervention (PCI), in coronary artery bypass grafting (CABG) patients.

**Methods and results:**

All patients who underwent the first time, isolated CABG in Sweden 2009–2020 and survived until discharge were included. The four-item PRECISE-DAPT score, based on age, estimated glomerular filtration rate, pre-operative haemoglobin concentration, and previous spontaneous bleeding, was calculated in patients discharged on DAPT (*n* = 6838), or antiplatelet monotherapy (*n* = 15 406). High bleeding risk was defined as a score ≥25 in accordance with previous studies and major bleeding as hospitalization due to bleeding. Associations were assessed by C-statistics and Cox regression models.

Major bleeding occurred during the first post-operative year in 130 patients (1.9%) in the DAPT group, and in 197 patients (1.3%) in the monotherapy group. The score identified 32.9% of the patients in the DAPT group and 38.2% in the monotherapy groups as having high bleeding risk. The area under the ROC-curve for the score was 0.67 (95%CI 0.62–0.72) for DAPT and 0.71 (0.67–0.74) for monotherapy. The hazard ratio for high bleeding risk vs. very low risk was 4.14 (2.07–8.26) for DAPT patients, and 4.95 (2.61–9.39) for monotherapy patients, both *P* < 0.001.

**Conclusion:**

The PRECISE-DAPT identifies patients with increased risk for major bleeding after discharge following CABG with moderate accuracy. The accuracy is comparable to what previously has been reported for patients after PCI.

## Graphical Abstract

**Graphical Abstract:**
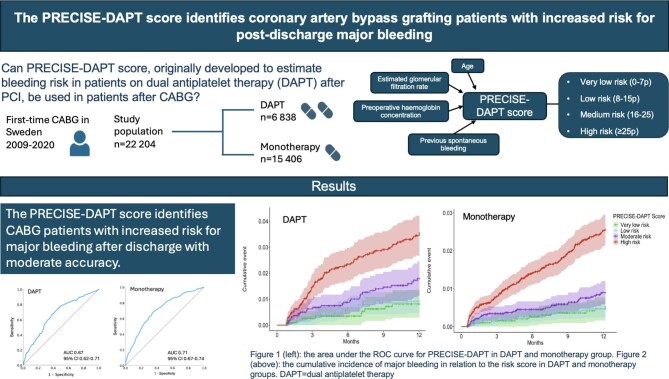
Figure 1 (left): the area under the ROC curve for PRECISE-DAPT in DAPT and monotherapy group. Figure 2 (above): the cumulative incidence of major bleeding in relation to the risk score in DAPT and monotherapy groups. DAPT, dual antiplatelet therapy.

## Introduction

Current guidelines recommend treatment with platelet inhibitors to reduce the risk for future ischaemic events in all patients undergoing coronary artery bypass grafting (CABG).^1–3^ Antiplatelet monotherapy, usually with acetylsalicylic acid (ASA), is recommended in patients with chronic coronary artery disease (CAD). In CAD patients presenting with acute coronary syndrome (ACS), dual antiplatelet therapy (DAPT) with ASA and a P2Y_12_-inhibitor, is recommended.^1–3^

Antiplatelet therapy is effective in reducing the risk for ischaemic events in CAD patients but increases the risk for bleeding complications. The bleeding risk is further enhanced with DAPT.^3^ If a major out-of-hospital bleeding event occurs, the mortality risk is markedly increased, as shown both in CABG patients^4^ and in other CAD populations.^5–8^

The bleeding risk varies markedly in patients treated with platelet inhibitors, depending on the potency of the antithrombotic treatment used and existing comorbidities. Among patients undergoing percutaneous coronary interventions (PCI), risk scores have been developed for the identification of patients with high post-discharge bleeding risk.^9–12^ In CABG patients, previous studies have focused on peri-operative bleeding risk and transfusion requirements, rather than on post-discharge bleeding. It remains unclear if post-discharge bleeding risk scores, originally developed to estimate bleeding risk in CAD patients undergoing PCI, also identify CABG patients with increased bleeding risk.

The PRECISE-DAPT score was originally developed to estimate the risk for post-discharge major bleeding, and was shown useful in guiding the duration of DAPT after PCI.^10^ The score has been extensively validated in various CAD populations that never have included CABG patients.^13–17^ Hence, the objective of the present study was to validate the PRECISE-DAPT score in a large nationwide cohort of CABG patients treated with platelet inhibitors.

## Methods

### Study design and study population

**Figure 1:**
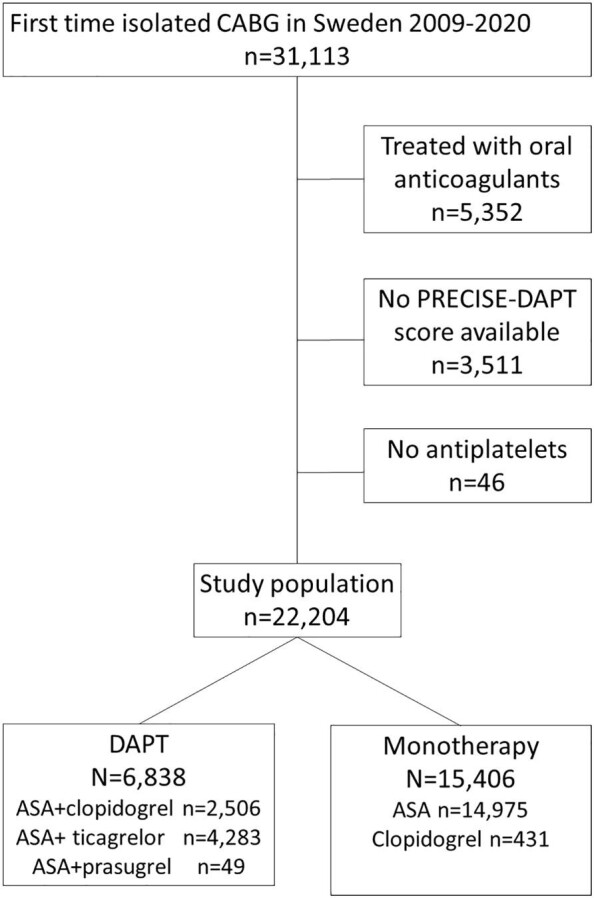
A flow chart showing inclusion and exclusion criteria for the study.

All patients >18 years old, who had undergone isolated, first time CABG in Sweden from 2009 to 2020, were included in an observational cohort study. The patients were identified in the Swedish Cardiac Surgery Register,^18^ which is a part of the SWEDEHEART registry.^19^ The CABG patients were divided into two groups. The DAPT group consisted of patients treated at discharge with ASA and a P2Y_12_-inhibitor (clopidogrel, ticagrelor, or prasugrel). The monotherapy group was CABG patients treated at baseline with either ASA or a P2Y_12_-inhibitor. Baseline was arbitrarily set 14 days after discharge to prevent the inclusion of bleeding events caused by the surgical procedure. Exclusion criteria were no treatment with platelet inhibitors, treatment with oral anticoagulants, and missing variables necessary to calculate the PRECISE-DAPT score. A flow chart with inclusion and exclusion criteria is presented in *Figure 1*.

The PRECISE-DAPT scores were derived from the patients’ clinical characteristics at baseline. Major bleeding was registered during the first 12 months after baseline. Major bleeding was defined as a hospitalization with a primary diagnosis of bleeding according to the International Classification of Diseases system (ICD-9, ICD-10) codes. The codes used are listed in the Supplementary material online, *Table S1*.

### Data sources

This study included data from five Swedish nationwide mandatory registers and databases. With help of the unique identification number that all Swedish citizens hold, the databases can be linked for research purposes. Baseline characteristics for the CABG procedure including pre-operative clinical data and post-operative complications were obtained from the Swedish Cardiac Surgery Registry, a part of the SWEDEHEART registry, which covers all open cardiac surgery procedures in Sweden since 1992.^18,19^ The National Patient Register,^20^ established in 1964, holds data on all hospital admission diagnoses and was used in conjunction with the Swedish Cardiac Surgery Registry to collect data on comorbidities and hospitalizations for major bleeding. The ICD-9 and ICD-10 codes used are listed in Supplementary material online, *Table S1*. The Swedish Prescribed Drug Register contains detailed information on every prescription dispensed from any Swedish pharmacy since July 2005.^21^ The register was used to collect data on dispensed prescriptions for platelet inhibitors and oral anticoagulants. The Anatomical Therapeutic Chemical codes used to identify the medications are listed in Supplementary material online, *Table S2*. The Swedish Cause of Death Register was used to collect data on mortality. Lastly, demographic data and emigration status was collected from the Swedish Population Register.

### PRECISE-DAPT

The PRECISE-DAPT was originally presented as a five-item (age, haemoglobin concentration, creatinine clearance, white-blood-cell count, and history of bleeding) bleeding risk score, to predict out-of-hospital bleeding in PCI patients on DAPT.^10^ The score was developed to guide clinicians about the optimal DAPT duration post-PCI, selecting high bleeding risk patients (score >25) for a shorter treatment (i.e. 3–6 months) and non–high-risk patients for prolonged treatment (i.e. ≥12 months). A simplified four-item version of the score, without white-blood-cell count, was also developed and validated.^10,22,23^ In the present study, the four-item score was computed due to no information on white-blood-cell count within our registry. Patients were classified into four risk categories: very low risk (score 0–7), low risk (score 8–15), moderate risk (score 16–24), and high risk (score ≥25).^22^ All data necessary for score calculations were prospectively collected in the registries.

### Ethics, reporting, and data sharing

This study was conducted according to the Declaration of Helsinki and was approved by the Swedish Ethical Review Authority (registration number 2021-00122, approved 31 March 2021). The need for individual patient consent was waived by the authority. This report adheres to The Strengthening the Reporting of Observational Studies in Epidemiology (STROBE) guidelines.^24^ The data used in this study will be shared upon reasonable request to the corresponding author if approval from the SWEDEHEART registry, the Swedish Ethical Review Authority, and the National Board of Health and Welfare have been received.

### Statistical analysis

Continuous variables are summarized as mean with standard deviation (SD) and categorical values are presented as frequencies with percentages. Comparisons between continuous variables were made using the Student's *t*-test and between categorical variables using chi-squared tests. Follow-up was set to 1 year after CABG. Bleeding rates were calculated in each risk group using the Kaplan-Meier method and compared using the log-rank test. To further compare different risk groups, the hazard ratio (HR) and a 95% confidence interval was calculated using Cox proportional hazard analysis. The proportional hazard assumption was tested using scaled Schoenfeld residuals and the ratio of hazards was constant over time.

Furthermore, to test the score's prognostic accuracy for 1-year bleeding, c-statistics derived from receiver operating characteristic (ROC) curves were used. Overall, a c-statistic <0.60 is considered to have poor discrimination, 0.60–0.75 possibly helpful discrimination and >0.75, clearly useful discrimination.^25^ For calibration, the observed proportion of major bleeding vs. the estimated probability of major bleeding ratio was calculated. The estimated probability was calculated according to the original description of the PRECISE-DAPT score.^10^ Youden's index, J = (sensitivity + specificity − 1), was calculated to determine the optimal cut-off level to identify high bleeding risk patients regarding sensitivity and specificity.

A *P*-value of <0.05 was considered statistically significant. Data was processed using IBM SPSS Statistics for Windows, version 28.0 (IBM Corp., Armonk, N.Y., USA) and R version 4.3.2 (R: A language and environment for statistical computing. R Foundation for Statistical Computing, Vienna, Austria).

## Results

### Patient cohorts

From 2009 to 2020, a total of 31 113 patients underwent first-time isolated CABG in Sweden. Complete data to calculate the PRECISE-DAPT score was available in 27 603/31 113 patients (88.7%). After exclusion of patients treated with oral anticoagulants post-operatively (*n* = 5352) and patients without either oral coagulants or platelet inhibitors (*n* = 46), 22 244 patients remained in the final study population. Out of these, 6838 (30.7%) patients were treated with DAPT and 15 406 (69.3%) were treated with antiplatelet monotherapy at discharge. A flow chart of included and excluded patients is presented in *Figure 1*. Baseline characteristics of patients in the DAPT and monotherapy groups are presented in *Table 1*. DAPT patients were younger, had better renal function, less often peripheral artery disease, atrial fibrillation, and less often a history of major bleeding. DAPT patients were more likely to suffer from prior myocardial infarction and PCI than the monotherapy group.

**Table 1:** Baseline characteristics of patients in the DAPT and monotherapy groups

	DAPT*n* = 6838	Monotherapy*n* = 15 406	*P*-value
Age, years	66.2 ± 9.3	68.1 ± 8.9	<0.001
Males	5607 (82.0)	12 580 (81.7)	0.54
Body mass index, kg/m^2^	27.8 ± 7.6	27.7 ± 7.1	0.41
Indication for CABG			<0.001
Acute coronary syndrome	5385 (78.8%)	8911 (57.8%)	
Chronic CAD	1453 (21.2)	6495 (42.2%)	
eGFR <60 mL/min	1014 (14.8)	2786 (18.1)	<0.001
Diabetes	2154 (31.5)	4919 (31.9)	0.53
Heart failure	1106 (16.2)	2449 (15.9)	0.60
Peripheral vascular disease	511 (7.5)	1498 (9.7)	<0.001
Atrial fibrillation	1204 (17.6)	3229 (21.0)	<0.001
Previous stroke	468 (6.8)	1142 (7.4)	0.13
Previous major bleeding	1580 (23.1)	3963 (25.7)	<0.001
Previous myocardial infarction	4990 (73.0)	6975 (45.3)	<0.001
Previous PCI	2031 (29.7)	2437 (15.8)	<0.001
Anaemia^a^	1344 (19.7)	3146 (20.4)	0.19

The results are reported as mean and standard deviation or number and percentage (%).

CABG, coronary artery bypass surgery; CAD, coronary artery disease; eGFR, estimated glomerular filtration rate; PCI, percutaneous coronary intervention.

a Anaemia was defined as haemoglobin level less than 13 g/dL in men and 12 g/dL in women.

### Bleeding risk

**Figure 2:**
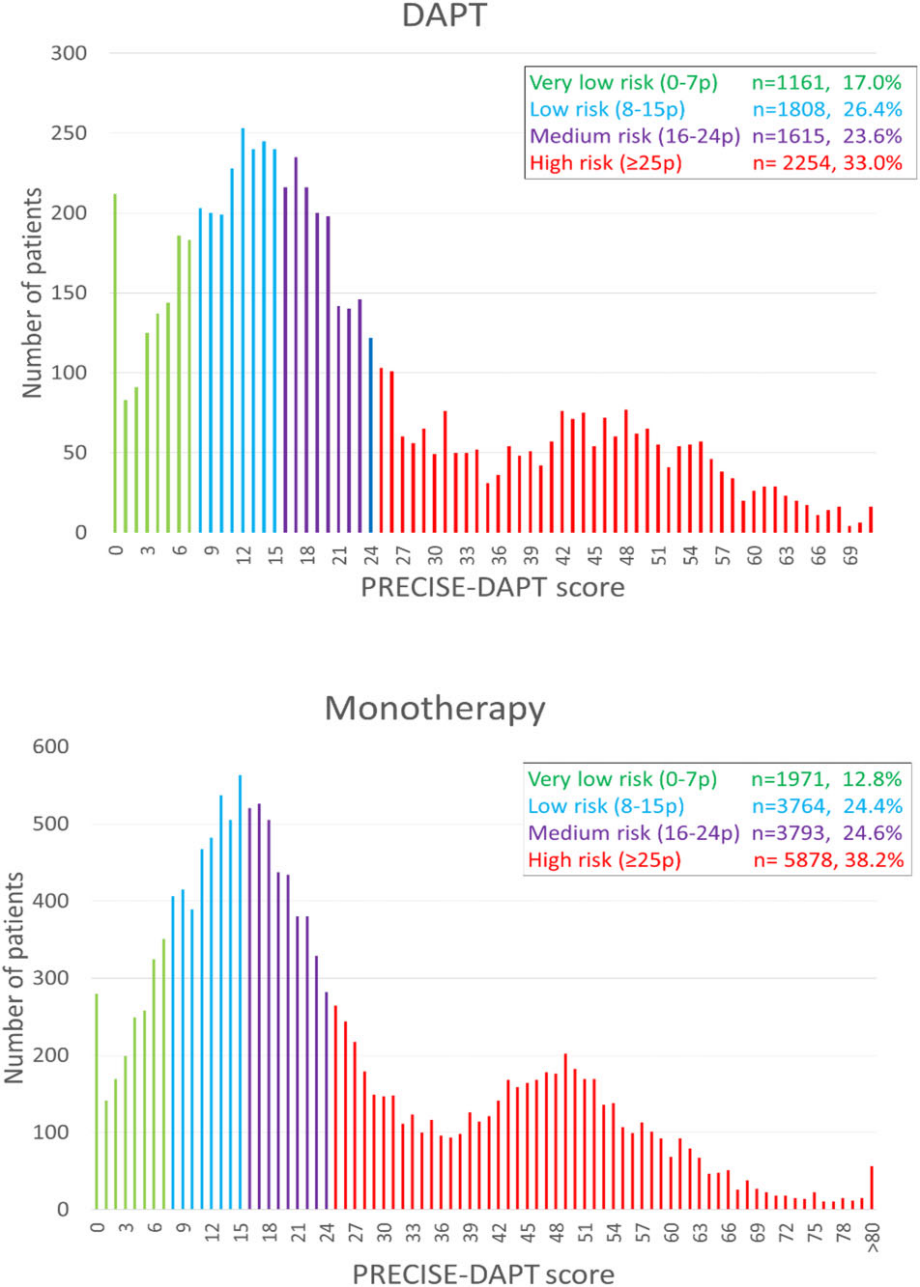
The distribution of the risk score in the DAPT and monotherapy groups.

Patient characteristics according to the bleeding risk group in the DAPT and the monotherapy groups are presented in Supplementary material online, *Tables S3* and S4. In the DAPT group, the mean PRECISE-DAPT score was 22.7 (SD 17.3, range 0–88) and the median score was 17 (25–75th percentile 10–32). In the monotherapy group, the mean PRECISE-DAPT score was 25.2 (SD 18.0, range 0–94) and the median score was 19 (25–75th percentile 12–38). According to PRECISE-DAPT, 33.0% of the patients in the DAPT group and 38.2% of the patients in the monotherapy group were at high risk for post-discharge major bleeding. The distribution of the risk score in the DAPT and monotherapy group is depicted in *Figure 2*.

**Figure 3:**
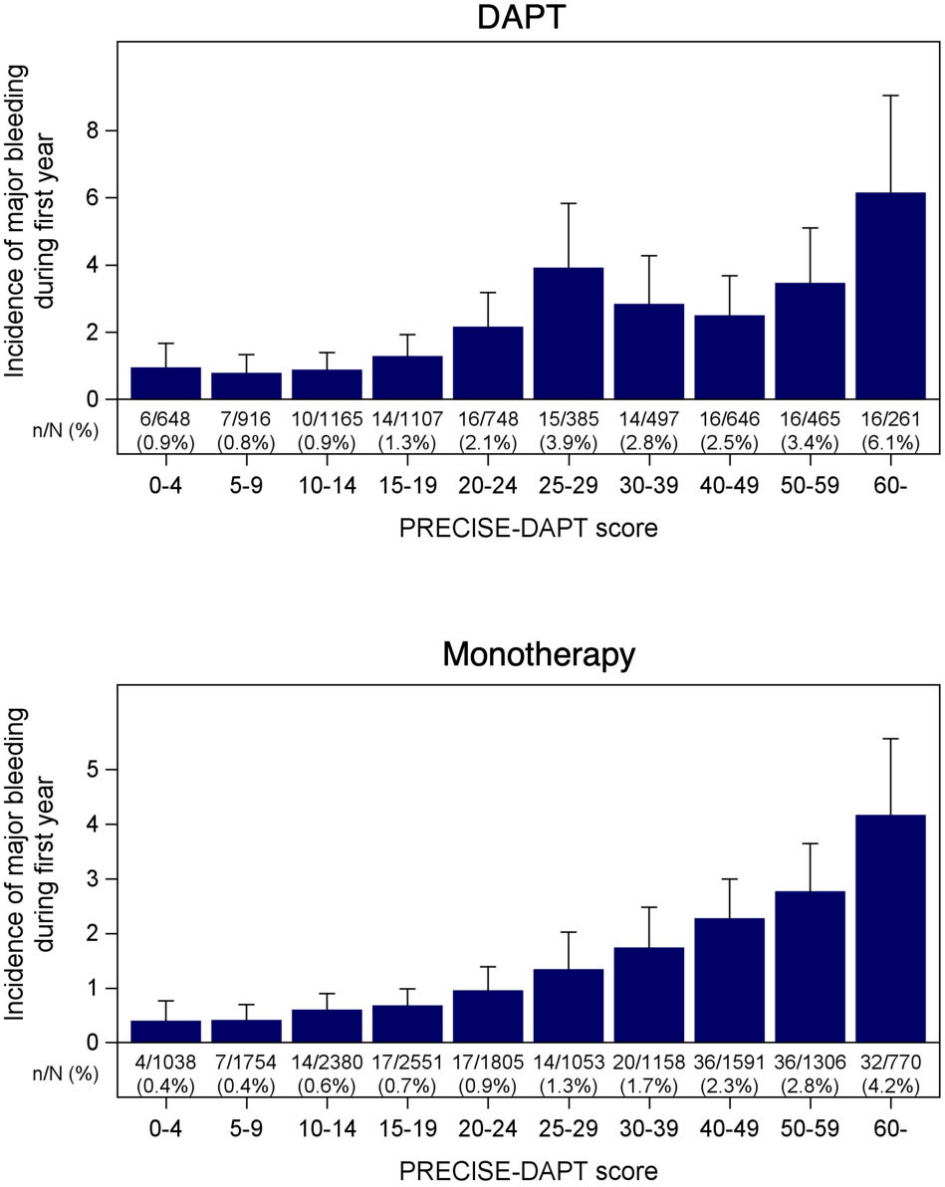
The incidence of major bleeding during the first year in relation to the risk score for patients in the DAPT and monotherapy groups.

**Figure 4:**
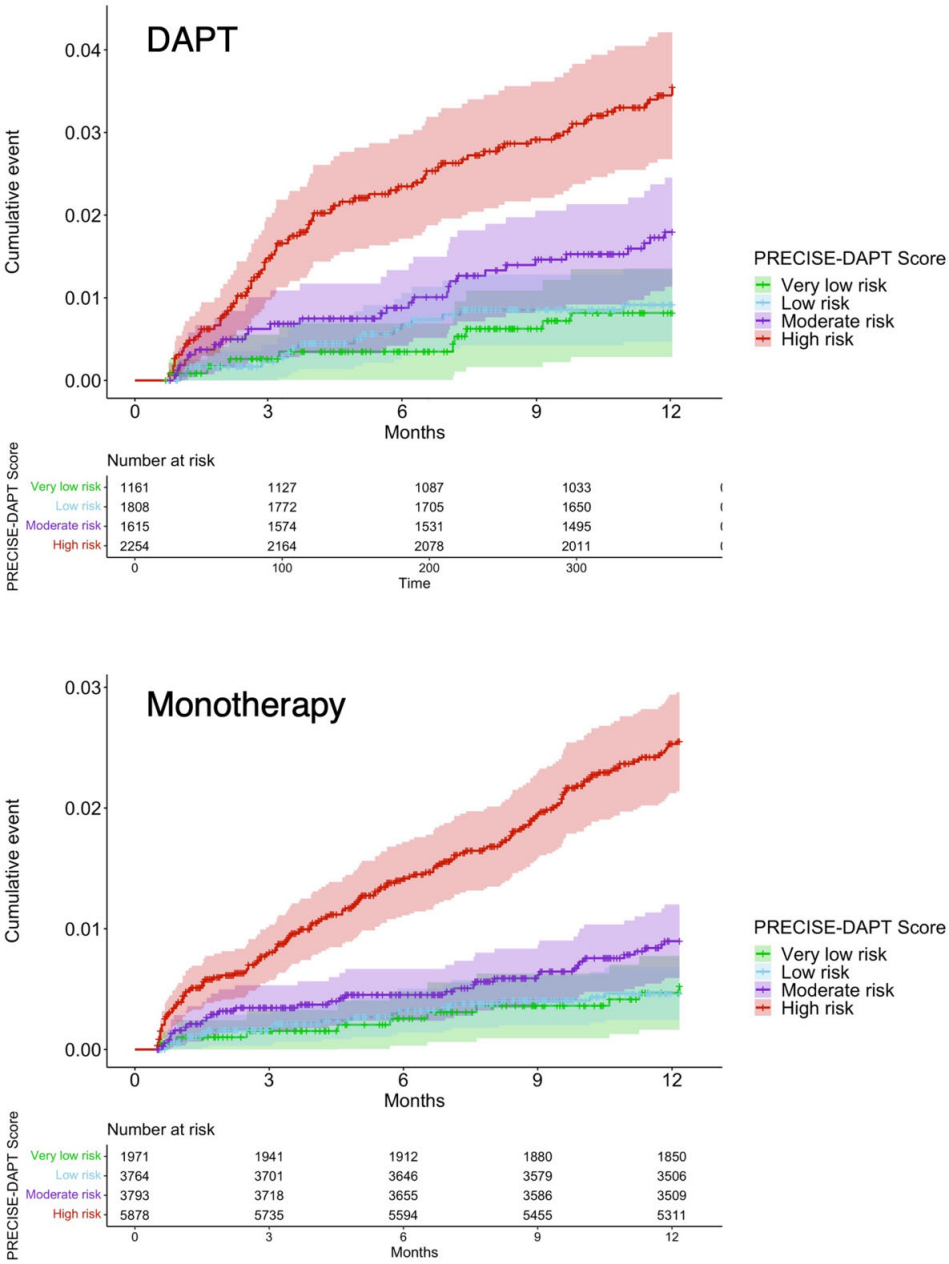
The cumulative incidence of major bleeding in relation to the risk score in DAPT and monotherapy groups.

A total of 327 patients (1.5%) experienced at least one major bleeding event during the first post-operative year, 130 patients in the DAPT group (1.9%) and 197 patients in the monotherapy group (1.3%). The incidences of major bleeding in relation to the risk score for patients in the DAPT and monotherapy groups are presented in *Table 2* and *Figure 3*. The incidence of bleeding was significantly higher in patients with higher than compared with lower bleeding risk. In the DAPT group, the incidence during the first 12 months was from 0.8%, 0.9%, 1.7%, and 3.4% respectively, in the very low, low, medium, and high-risk groups. The corresponding figures for the monotherapy group were 0.5%, 0.5%, 0.9%, and 2.3% respectively. The HR for the high-risk group vs. the lowest risk group was 4.14 (2.07–8.26) for DAPT patients, and 4.95 (2.61–9.39) for monotherapy patients, both *P* < 0.001 (*Table 2* and *Figure 4*).

**Table 2:** Number of patients in the DAPT and monotherapy groups in relation to their risk score

	DAPT			Monotherapy		
	Number of patients (%)	Number of events	Hazard ratio and 95% CI and *P*-value	Number of patients (%)	Number of events	Hazard ratio and 95% CI and *P*-value
PRECISE-DAPT risk group						
Very low (0–7 points)	1161 (17.0%)	9 (0.8%)	1 (reference)	1971 (12.8%)	9 (0.5%)	1 (reference)
Low (8–15 points)	1808 (26.4%)	16 (0.9%)	1.11 (0.50–2.52) *P* = 0.80	3764 (24.4%)	17 (0.5%)	0.89 (0.41–1.95) *P* = 0.77
Moderate (16–24 points)	1615 (23.6%)	28 (1.7%)	2.20 (1.0–4.67) *P* = 0.039	3793 (24.6%)	33 (0.9%)	1.72 (0.85–3.50) *P* = 0.13
High (≥25 points)	2254 (33.0%)	77 (3.4%)	4.14 (2.07–8.26) *P* < 0.001	5878 (38.2%)	138 (2.3%)	4.95 (2.61–9.39) *P* < 0.001
All	6838 (100%)	130 (1.9%)		15 406 (100%)	197 (1.3%)	

### Discriminatory accuracy and calibration

**Figure 5:**
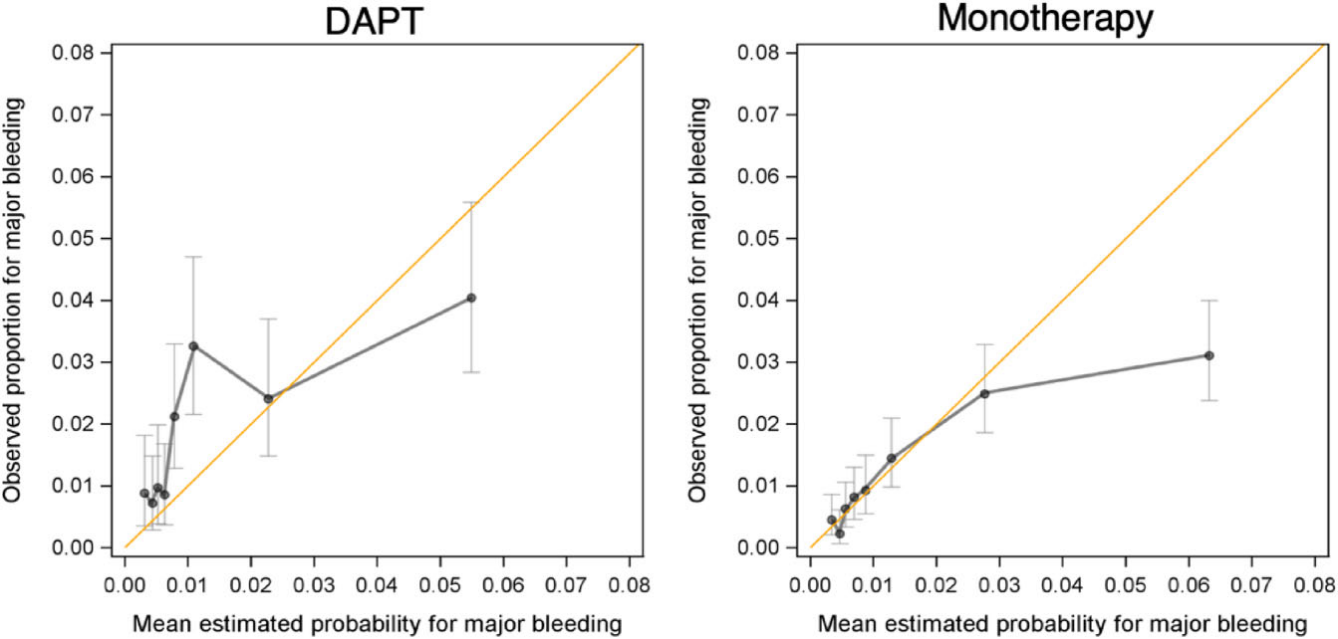
The O/E ratios for DAPT and monotherapy groups are presented.

The area under the ROC curve for PRECISE-DAPT was 0.67 (95%CI 0.62–0.71) in the DAPT group, and 0.71 (95%CI 0.67–0.74) in the monotherapy group, Supplementary material online, *Figure S1*. The observed and estimated number of major bleeding events were 130 and 98, respectively, in the DAPT group (O/E ratio 1.32), and 197 and 256, respectively, in the monotherapy group (O/E ratio 0.77). The O/E ratios for DAPT and monotherapy groups are presented in *Figure 5*. The score underestimated the bleeding risk in low-risk DAPT patients and overestimated the risk in high-risk monotherapy patients.

According to Youden's index, a PRECISE-DAPT cut off level of ≥22 points for patients on DAPT and ≥27 points for monotherapy patients gave the optimal combination of sensitivity and specificity. In patients on DAPT, the change from the original cut off level of 25 to 22 points increased sensitivity from 59 to 67% but reduced specificity from 68 to 61%. In patients with monotherapy, the change to 27 points reduced sensitivity from 70 to 68% while specificity increased from 63 to 66%.

## Discussion

The main findings of this large nationwide cohort study were, (1) The PRECISE-DAPT score showed moderate discrimination for major bleeding in patients treated with CABG, which is in line with prior reports of patients undergoing PCI. (2) The score underestimated the bleeding risk in CABG patients initially treated with DAPT and overestimated the risk in patients treated with antiplatelet monotherapy.

There is scarce knowledge about the incidence and consequences of post-discharge bleeding in CABG patients. Our group reported recently that during a median follow-up of 6.0 years, 6.6% of CABG patients experienced a major bleeding event after discharge.^4^ In that study, partly based on the same study population as in the present study, major bleeding was associated with markedly increased mortality risk, especially early after the bleeding event. In our CABG population, the frequency and the mortality risk of major bleeding were comparable to the corresponding figures for myocardial infarction.^4^ These observations are consistent with similar findings in the PCI population.^26^

DAPT reduces the risk for ischaemic events in patients with ACS but increases the risk for bleeding.^3^ The PRECISE-DAPT score was developed to identify patients with high bleeding risk after stent implantation and DAPT treatment.^10^ In patients treated with PCI, the score can be used to support clinical decision-making about DAPT treatment duration. The score identifies PCI patients in whom the benefits of prolonged DAPT treatment outweighs the risks and vice versa.^10^ The score has been validated in different PCI populations but not in CABG patients. In a recent meta-analysis, based on 21 studies with a total of 67 283 PCI patients, the external validity of the score was confirmed and identified 24.7% of the patients at high bleeding risk.^15^

In the present study, the PRECISE score identified 32.9% of the patients in the DAPT group and 38.2% of the patients in the monotherapy group as high risk for major bleeding. These figures are higher than in PCI-patients in the aforementioned meta-analysis (24.7%),^15^ suggesting that CABG patients may have a higher bleeding risk than PCI patients. Interestingly, despite the higher proportion of CABG patients treated with DAPT with a high PRECISE-DAPT score, the incidence of major bleeding at 12 months was comparable in the present study (1.9%) to what has previously been reported in PCI-DAPT patients (2.0%).^10,15^ The reason for this is unclear but may depend on the duration of the treatment and/or different definitions of major bleeding. Furthermore, the predictive accuracy of the score, as measured with c-statistics, in CABG patients (area under the ROC curve 0.67 in DAPT and 0.71 in monotherapy patients) was comparable to the accuracy previously reported in PCI patients.^15^

We calculated the optimal PRECISE-DAPT cut-off levels for high bleeding risk in patients treated with DAPT or monotherapy. Using these cut-offs instead of the original had limited impact on sensitivity and specificity. Hence, the original cut-off of 25 points to classify high bleeding risk appears applicable also in CABG patients.

A bleeding risk score, which is shown useful in CABG patients and in other cardiac surgery patient populations, might serve different purposes in the future. This may include an informed decision-making on the number of antiplatelet agents, their association with oral anticoagulation in patients with new-onset post-operative atrial fibrillation, or the type and duration of antithrombotic medication after valve repair or bioprosthetic valve implantation.

## Strengths and limitations

The present study has both strengths and limitations. Strengths include the large study population and the use of data from validated registries. No patients were lost to follow-up. Limitations include that standardized bleeding definitions, such as the Bleeding Academic Research Consortium bleeding classification or the Thrombolysis in Myocardial Infarction bleeding criteria, commonly used in clinical trials, could not be used since we did not have data on transfusions and haemoglobin levels. Even though not directly comparable to other definitions, we argue that our definition of major bleeding (hospitalization with a primary diagnosis of bleeding) is relevant from a clinical and epidemiological perspective. It is difficult to speculate whether the present definition has an impact on the overall incidence of post-discharge bleeding compared to the established definitions and if the choice of definition impacts the accuracy obtained with ROC curves. Furthermore, other tools and definitions of high bleeding risk, like Academic Research Consortium high bleeding risk criteria^27^ could not be used due to lack of information in the registries used. The division of patients into DAPT and monotherapy was correct at discharge but may change during the 1-year follow-up period, due to patients stopping taking their medications. Hence, the present study should be considered as a first attempt to use a tool for bleeding risk assessment in CABG patients. Future studies, preferably prospective, are warranted to evaluate other scores and criteria in CABG patients and to compare different tools.

## Conclusion

In conclusion, the PRECISE-DAPT score identifies with moderate accuracy CABG patients with increased post-discharge bleeding risk. The accuracy is comparable to previous findings in PCI-patients. PRECISE-DAPT may prove useful to inform on risks of intensified and/or prolonged antithrombotic treatment after CABG.

## Supplementary Material

pvae060_Supplemental_File

## Data Availability

The data underlying this article will be shared on reasonable request to the corresponding author.

## References

[1] Kulik A, Ruel M, Jneid H, Ferguson TB, Hiratzka LF, Ikonomidis JS, Lopez-Jimenez F, Mcnallan SM, Patel M, Roger VL, Sellke FW, Sica DA, Zimmerman L. Secondary prevention after coronary artery bypass graft surgery: a scientific statement from the American Heart Association. Circulation 2015;131:927–964. 25679302 10.1161/CIR.0000000000000182

[2] Sousa-Uva M, Head SJ, Milojevic M, Collet J-P, Landoni G, Castella M, Dunning J, Gudbjartsson T, Linker NJ, Sandoval E, Thielmann M, Jeppsson A, Landmesser U. 2017 EACTS guidelines on perioperative medication in adult cardiac surgery. Eur J Cardiothorac Surg 2018;53:5–33. 29029110 10.1093/ejcts/ezx314

[3] Valgimigli M, Bueno H, Byrne RA, Collet JP, Costa F, Jeppsson A, Jüni P, Kastrati A, Kolh P, Mauri L, Montalescot G, Neumann FJ, Petricevic M, Roffi M, Steg PG, Windecker S, Zamorano JL, Levine GN, ESC Scientific Document Group. 2017 ESC focused update on dual antiplatelet therapy in coronary artery disease developed in collaboration with EACTS: the Task Force for dual antiplatelet therapy in coronary artery disease of the European Society of Cardiology (ESC) and of the European Association for Cardio-Thoracic Surgery (EACTS). Eur Heart J 2018;39:213–260. 28886622 10.1093/eurheartj/ehx419

[4] Björklund E, Enström P, Nielsen SJ, Tygesen H, Martinsson A, Hansson EC, Lindgren M, Malm CJ, Pivodic A, Jeppsson A. Postdischarge major bleeding, myocardial infarction, and mortality risk after coronary artery bypass grafting. Heart 2024;110:569–577. 38148160 10.1136/heartjnl-2023-323394 PMC10982629

[5] Généreux P, Giustino G, Witzenbichler B, Weisz G, Stuckey TD, Rinaldi MJ, Neumann F-J, Metzger DC, Henry TD, Cox DA, Duffy PL, Mazzaferri E, Yadav M, Francese DP, Palmerini T, Kirtane AJ, Litherland C, Mehran R, Stone GW. Incidence, predictors, and impact of post-discharge bleeding after percutaneous coronary intervention. J Am Coll Cardiol 2015;66:1036–1045. 26314532 10.1016/j.jacc.2015.06.1323

[6] Jacobsen MR, Jabbari R, Engstrøm T, Grove EL, Glinge C, Pedersen F, Holmvang L, Køber L, Torp-Pedersen C, Maeng M, Veien K, Freeman P, Charlot MG, Kelbæk H, Sørensen R. Bleeding risk and P2Y12 inhibitors in all-comer patients with ST-segment elevation myocardial infarction treated with percutaneous coronary intervention: a single-centre cohort study. Eur Heart J Cardiovasc Pharmacother 2023;9:617–626. 37403404 10.1093/ehjcvp/pvad048

[7] Kazi DS, Leong TK, Chang TI, Solomon MD, Hlatky MA, Go AS. Association of spontaneous bleeding and myocardial infarction with long-term mortality after percutaneous coronary intervention. J Am Coll Cardiol 2015;65:1411–1420. 25857906 10.1016/j.jacc.2015.01.047

[8] Marquis-Gravel G, Dalgaard F, Jones AD, Lokhnygina Y, James SK, Harrington RA, Wallentin L, Steg PG, Lopes RD, Storey RF, Goodman SG, Mahaffey KW, Tricoci P, White HD, Armstrong PW, Ohman EM, Alexander JH, Roe MT. Post-discharge bleeding and mortality following acute coronary syndromes with or without PCI. J Am Coll Cardiol 2020;76:162–171. 32646565 10.1016/j.jacc.2020.05.031

[9] Abu-Assi E, Raposeiras-Roubin S, Cobas-Paz R, Caneiro-Queija B, Martínez-Reglero C, Rodríguez-Rodríguez JM, Baz A, Íñiguez-Romo A. Assessing the performance of the PRECISE-DAPT and PARIS risk scores for predicting one-year out-of-hospital bleeding in acute coronary syndrome patients. EuroIntervention 2018;13:1914–1922. 29131804 10.4244/EIJ-D-17-00550

[10] Costa F, Van Klaveren D, James S, Heg D, Räber L, Feres F, Pilgrim T, Hong M-K, Kim H-S, Colombo A, Steg PG, Zanchin T, Palmerini T, Wallentin L, Bhatt DL, Stone GW, Windecker S, Steyerberg EW, Valgimigli M. Derivation and validation of the predicting bleeding complications in patients undergoing stent implantation and subsequent dual antiplatelet therapy (PRECISE-DAPT) score: a pooled analysis of individual-patient datasets from clinical trials. Lancet 2017;389:1025–1034. 28290994 10.1016/S0140-6736(17)30397-5

[11] Lyu S-Q, Zhu J, Wang J, Wu S, Zhang H, Shao X-H, Yang Y-M. Performance of the REACH, PARIS, BleeMACS, and PRECISE-DAPT scores for predicting 1-year bleeding events in patients undergoing coronary drug-eluting stent implantation. Platelets 2022;33:719–726. 34634980 10.1080/09537104.2021.1981847

[12] Montalto C, Crimi G, Morici N, Piatti L, Grosseto D, Sganzerla P, Tortorella G, De Rosa R, De Luca L, De Luca G, Palmerini T, Valgimigli M, Savonitto S, De Servi S. Bleeding risk prediction in elderly patients managed invasively for acute coronary syndromes: external validation of the PRECISE-DAPT and PARIS scores. Int J Cardiol 2021;328:22–28. 33279593 10.1016/j.ijcard.2020.11.065

[13] Castelijns MC, Hageman SHJ, Teraa M, Van Der Meer MG, Westerink J, Costa F, Ten Berg JM, Visseren FLJ. External validation of bleeding risk models for the prediction of long-term bleeding risk in patients with established cardiovascular disease. Am Heart J 2023;260:72–81. 36841319 10.1016/j.ahj.2023.02.011

[14] Choi KH, Song YB, Lee JM, Park TK, Yang JH, Choi J-H, Choi S-H, Oh J-H, Cho D-K, Lee JB, Doh J-H, Kim S-H, Jeong J-O, Bae J-H, Kim B-O, Cho JH, Suh I-W, Kim D-I, Park H-K, Park J-S, Choi WG, Lee WS, Gwon H-C, Hahn J-Y. Clinical usefulness of PRECISE-DAPT score for predicting bleeding events in patients with acute coronary syndrome undergoing percutaneous coronary intervention: an analysis from the SMART-DATE randomized trial. Circ Cardiovasc Interv 2020;13:e008530. 32354228 10.1161/CIRCINTERVENTIONS.119.008530

[15] Munafò AR, Montalto C, Franzino M, Pistelli L, Di Bella G, Ferlini M, Leonardi S, D'ascenzo F, Gragnano F, Oreglia JA, Oliva F, Ortega-Paz L, Calabrò P, Angiolillo DJ, Valgimigli M, Micari A, Costa F. External validity of the PRECISE-DAPT score in patients undergoing PCI: a systematic review and meta-analysis. Eur Heart J Cardiovasc Pharmacother 2023;9:709–721. 37634083 10.1093/ehjcvp/pvad063

[16] Costa F, Valgimigli M, Steg PG, Bhatt DL, Hohnloser SH, Ten Berg JM, Miede C, Nordaby M, Lip GYH, Oldgren J, Cannon CP. Antithrombotic therapy according to baseline bleeding risk in patients with atrial fibrillation undergoing percutaneous coronary intervention: applying the PRECISE-DAPT score in RE-DUAL PCI. Eur Heart J Cardiovasc Pharmacother 2022;8:216–226. 33258897 10.1093/ehjcvp/pvaa135

[17] Gragnano F, Heg D, Franzone A, Mcfadden EP, Leonardi S, Piccolo R, Vranckx P, Branca M, Serruys PW, Benit E, Liebetrau C, Janssens L, Ferrario M, Zurakowski A, Diletti R, Dominici M, Huber K, Slagboom T, Buszman P, Bolognese L, Tumscitz C, Bryniarski K, Aminian A, Vrolix M, Petrov I, Garg S, Naber C, Prokopczuk J, Hamm C, Steg PG, Jüni P, Windecker S, Valgimigli M. PRECISE-DAPT score for bleeding risk prediction in patients on dual or single antiplatelet regimens: insights from the GLOBAL LEADERS and GLASSY. Eur Heart J Cardiovasc Pharmacother 2022;8:28–38. 32941620 10.1093/ehjcvp/pvaa106

[18] Vikholm P, Ivert T, Nilsson J, Holmgren A, Freter W, Ternström L, Ghaidan H, Sartipy U, Olsson C, Granfeldt H, Ragnarsson S, Friberg Ö. Validity of the Swedish Cardiac surgery registry. Interact Cardiovasc Thorac Surg 2018;27:67–74. 29452368 10.1093/icvts/ivy030

[19] Jernberg T, Attebring MF, Hambraeus K, Ivert T, James S, Jeppsson A, Lagerqvist B, Lindahl B, Stenestrand U, Wallentin L. The Swedish web-system for enhancement and development of evidence-based care in heart disease evaluated according to recommended therapies (SWEDEHEART). Heart 2010;96:1617–1621. 20801780 10.1136/hrt.2010.198804

[20] Ludvigsson JF, Andersson E, Ekbom A, Feychting M, Kim J-L, Reuterwall C, Heurgren M, Olausson PO. External review and validation of the Swedish national inpatient register. BMC Public Health 2011;11:450. 21658213 10.1186/1471-2458-11-450 PMC3142234

[21] Wettermark B, Hammar N, Michaelfored C, Leimanis A, Otterblad Olausson P, Bergman U, Persson I, Sundström A, Westerholm B, Rosén M. The new Swedish Prescribed Drug Register—opportunities for pharmacoepidemiological research and experience from the first six months. Pharmacoepidemiol Drug Saf 2007;16:726–735. 16897791 10.1002/pds.1294

[22] Costa F, Van Klaveren D, Colombo A, Feres F, Räber L, Pilgrim T, Hong M-K, Kim H-S, Windecker S, Steyerberg EW, Valgimigli M. A 4-item PRECISE-DAPT score for dual antiplatelet therapy duration decision-making. Am Heart J 2020;223:44–47. 32151822 10.1016/j.ahj.2020.01.014

[23] Wester A, Mohammad MA, Olivecrona G, Holmqvist J, Yndigegn T, Koul S. Validation of the 4-item PRECISE-DAPT score: a SWEDEHEART study. J Am Heart Assoc 2021;10:e020974. 34612051 10.1161/JAHA.121.020974 PMC8751860

[24] Von Elm E, Altman DG, Egger M, Pocock SJ, Gøtzsche PC, Vandenbroucke JP. The strengthening the reporting of Observational Studies in Epidemiology (STROBE) statement: guidelines for reporting observational studies. Lancet 2007;370:1453–1457. 18064739 10.1016/S0140-6736(07)61602-X

[25] Alba AC, Agoritsas T, Walsh M, Hanna S, Iorio A, Devereaux PJ, Mcginn T, Guyatt G. Discrimination and calibration of clinical prediction models: users' guides to the medical literature. JAMA 2017;318:1377–1384. 29049590 10.1001/jama.2017.12126

[26] Valgimigli M, Costa F, Lokhnygina Y, Clare RM, Wallentin L, Moliterno DJ, Armstrong PW, White HD, Held C, Aylward PE, Van de Werf F, Harrington RA, Mahaffey KW, Tricoci P. Trade-off of myocardial infarction vs. bleeding types on mortality after acute coronary syndrome: lessons from the Thrombin Receptor Antagonist for Clinical event Reduction in Acute Coronary Syndrome (TRACER) randomized trial. Eur Heart J 2017;38:804–810. 28363222 10.1093/eurheartj/ehw525 PMC5837470

[27] Urban P, Mehran R, Colleran R, Angiolillo DJ, Byrne RA, Capodanno D, Cuisset T, Cutlip D, Eerdmans P, Eikelboom J, Farb A, Gibson CM, Gregson J, Haude M, James SK, Kim H-S, Kimura T, Konishi A, Laschinger J, Leon MB, Magee PFA, Mitsutake Y, Mylotte D, Pocock S, Price MJ, Rao SV, Spitzer E, Stockbridge N, Valgimigli M, Varenne O, Windhoevel U, Yeh RW, Krucoff MW, Morice M-C. Defining high bleeding risk in patients undergoing percutaneous coronary intervention. Circulation 2019;140:240–261. 31116032 10.1161/CIRCULATIONAHA.119.040167 PMC6636810

