# Supplementary material for: The four-item PRECISE-DAPT score identifies coronary artery bypass grafting patients with increased risk for post-discharge major bleeding
Source: Eur Heart J Cardiovasc Pharmacother. 2024 Aug 20;11(1):48–56. doi: 10.1093/ehjcvp/pvae060 (PMC11805682; doi:10.1093/ehjcvp/pvae060)

Supplementary material to:

**The PRECISE-DAPT score identifies coronary artery bypass grafting patients with increased risk for post-discharge major bleeding**

Philip Enström^1,2^, Andreas Martinsson^2,3^, Mary Rezk^1,2^, Susanne Nielsen^1,2^, Erik Björklund^2,4^, Maya Landenhed-Smith^1,2^, Emily Pan^5^, Anders Jeppsson^1,2^

1. Department of Cardiothoracic Surgery, Sahlgrenska University Hospital, Gothenburg, Sweden;
2. Department of Molecular and Clinical Medicine, Institute of Medicine, Sahlgrenska Academy, University of Gothenburg, Gothenburg, Sweden;
3. Department of Cardiology, Sahlgrenska University Hospital, Gothenburg, Sweden;
4. Department of Medicine, Southern Älvborg Hospital, Borås, Sweden;
5. Department of Surgery, Central Finland Hospital Nova, Jyväskylä, Finland

**Supplementary Table S1: The 9^th^ and 10^th^ ICD-codes used in the study.**

|  | **ICD-9** | **ICD-10** |
| --- | --- | --- |
| Diabetes | 250 | E10-E14 |
| Heart failure | 425, 428 | I42, I43, I50 |
| Peripheral vascular disease | 440-444 | I70-I74, I77 |
| Atrial fibrillation | 427D | I48 |
| Stroke | 434, 436 | I61-I64, I69 |
| Bleeding | 280, 282, 285-287, 362W, 430-432, 530C, 531-533, 578A-B, 599H, 626G, 626W, 626X, 719B, 784H, 784W, 786D | D50, D62, D68-D69, H356, H922, I230, I312, I60-I62, I690-I692,I850, I983, J942, K221, K226, K25-K28, K290, K625, K661, K920, K921, K922, M250, N02, N398, N421, N501A, N939, N950, R31, R040-R042, R048, R049 |
| Myocardial infarction | 410 | I21 |

**Supplementary Table S2.** ATC-codes used for defining pharmacotherapies.

|  | **ATC-codes** |
| --- | --- |
| **Acetylsalicylic acid** | B01AC06 |
| **Clopidogrel** | B01AC04 |
| **Prasugrel** | B01AC22 |
| **Ticagrelor** | B01AC24 |
| **Oral anticoagulants** | B01AA03, B01AE07, B01AF01, B01AF02, B01AF03 |

**Supplementary Table S3.** The baseline characteristics of DAPT patients according to their bleeding risk group. The results are reported as mean and standard deviation or number and percentage (%).

|  | Very low risk n=1161 | Low risk n=1808 | Medium risk n=1615 | High risk n=2254 | p-value |
| --- | --- | --- | --- | --- | --- |
| Age (years) | 54.2±6.2 | 64.4±5.7 | 71.1±5.9 | 60.3±9.0 | <0.001 |
| Males | 1035 (18.5%) | 1569 (28.0%) | 1327 (23.7%) | 1676 (29.9%) | <0.001 |
| Body Mass Index (kg/m^2^) | 28.6±4.3 | 28.0±11.0 | 27.5±8.4 | 27.4±4.2 | <0.001 |
| Indication for CABG |  |  |  |  | 0.029 |
| Acute coronary syndrome | 902 (16.8%) | 1387 (25.8%) | 1291 (24.0%) | 1805 (26.4%) |  |
| Chronic CAD | 259 (17.8%) | 421 (29.0%) | 324 (22.3%) | 449 (30.9%) |  |
| eGFR <60 ml/min | 0 (0.0%) | 5 (0.5%) | 175 (17.3%) | 834 (82.2%) | <0.001 |
| Diabetes | 356 (16.5%) | 530 (24.6%) | 488 (22.7%) | 780 (36.2%) | 0.001 |
| Heart failure | 126 (11.4%) | 239 (21.6%) | 249 (22.5%) | 492 (44.5%) | <0.001 |
| Peripheral vascular disease | 28 (5.5%) | 80 (15.7%) | 141 (27.6%) | 262 (51.3%) | <0.001 |
| Atrial fibrillation | 130 (10.8%) | 284 (23.6%) | 320 (26.6%) | 470 (39.0%) | <0.001 |
| Previous stroke | 27 (5.8%) | 82 (17.5%) | 109 (23.3%) | 250 (53.4%) | <0.001 |
| Previous major bleeding | 0 (0.0%) | 0 (0.0%) | 0 (0.0%) | 1580 (100%) | <0.001 |
| Previous myocardial infarction | 836 (16.8%) | 1277 (25.6%) | 1174 (23.5%) | 1703 (34.1%) | 0.004 |
| Previous PCI | 366 (18.0%) | 528 (26.0%) | 471 (23.2%) | 666 (32.8%) | 0.510 |
| Anemia* | 72 (5.4%) | 203 (15.1%) | 275 (20.5%) | 794 (59.1%) | <0.001 |

CABG = coronary artery bypass surgery, CAD = coronary artery disease, eGFR = estimated glomerular filtration rate, PCI = percutaneous coronary intervention. *Anemia defined as hemoglobin level less than 13 g/dl in men and 12 g/dl in women.

**Supplementary Table S5. The baseline characteristics of monotherapy patients according to their bleeding risk group**. The results are reported as mean and standard deviation or number and percentage (%).

|  | Very low risk  n=1971 | Low risk  n=3764 | Medium risk  n=3793 | High risk  n=5878 | p-value |
| --- | --- | --- | --- | --- | --- |
| Age (years) | 55.1±5.7 | 65.1±5.4 | 71.9±5.8 | 71.8±8.4 | <0.001 |
| Males | 1730 (13.8%) | 3301 (26.2%) | 3117 (24.8%) | 4432 (35.2%) | <0.001 |
| Body Mass Index (kg/m^2^) | 28.6±5.2 | 27.9±6.3 | 27.4±8.1 | 27.6±7.4 | <0.001 |
| Indication for CABG |  |  |  |  | <0.001 |
| Acute coronary syndrome | 1130 (12.7%) | 2036 (22.8%) | 2064 (23.2%) | 3681 (41.3%) |  |
| Chronic CAD | 841 (12.9%) | 1728 (26.6%) | 1729 (26.6%) | 2197 (33.8%) |  |
| eGFR <60 ml/min | 0 (0.0%) | 8 (0.3%) | 352 (12.6%) | 2426 (87.1%) | <0.001 |
| Diabetes | 639 (13.0%) | 1057 (21.5%) | 1035 (21.0%) | 2188 (44.5%) | <0.001 |
| Heart failure | 214 (8.7%) | 440 (18.0%) | 502 (20.5%) | 1293 (52.8%) | <0.001 |
| Peripheral vascular disease | 66 (4.4%) | 226 (15.1%) | 357 (23.8%) | 849 (56.7%) | <0.001 |
| Atrial fibrillation | 171 (5.3%) | 631 (19.5%) | 846 (26.2%) | 1581 (49.0%) | <0.001 |
| Previous stroke | 49  (4.3%) | 165 (14.4%) | 252 (22.1%) | 676 (59.2%) | <0.001 |
| Previous major bleeding | 0 (0.0%) | 0 (0.0%) | 0 (0.0%) | 3963 (100%) | <0.001 |
| Previous myocardial infarction | 833 (11.9%) | 1519 (21.8%) | 1587 (22.8%) | 3036 (43.5%) | <0.001 |
| Previous PCI | 340 (14.0%) | 560 (23.0%) | 546 (22.4%) | 991 (40.7%) | <0.001 |
| Anemia* | 139 (4.4%) | 353 (11.2%) | 598 (19.0%) | 2056 (65.4%) | <0.001 |

CABG = coronary artery bypass surgery, CAD = coronary artery disease, eGFR = estimated glomerular filtration rate, PCI = percutaneous coronary intervention. *Anemia defined as hemoglobin level less than 13 g/dl in men and 12 g/dl in women.

Supplementary Fig S1. The area under the ROC curve for PRECISE-DAPT in DAPT and monotherapy group.


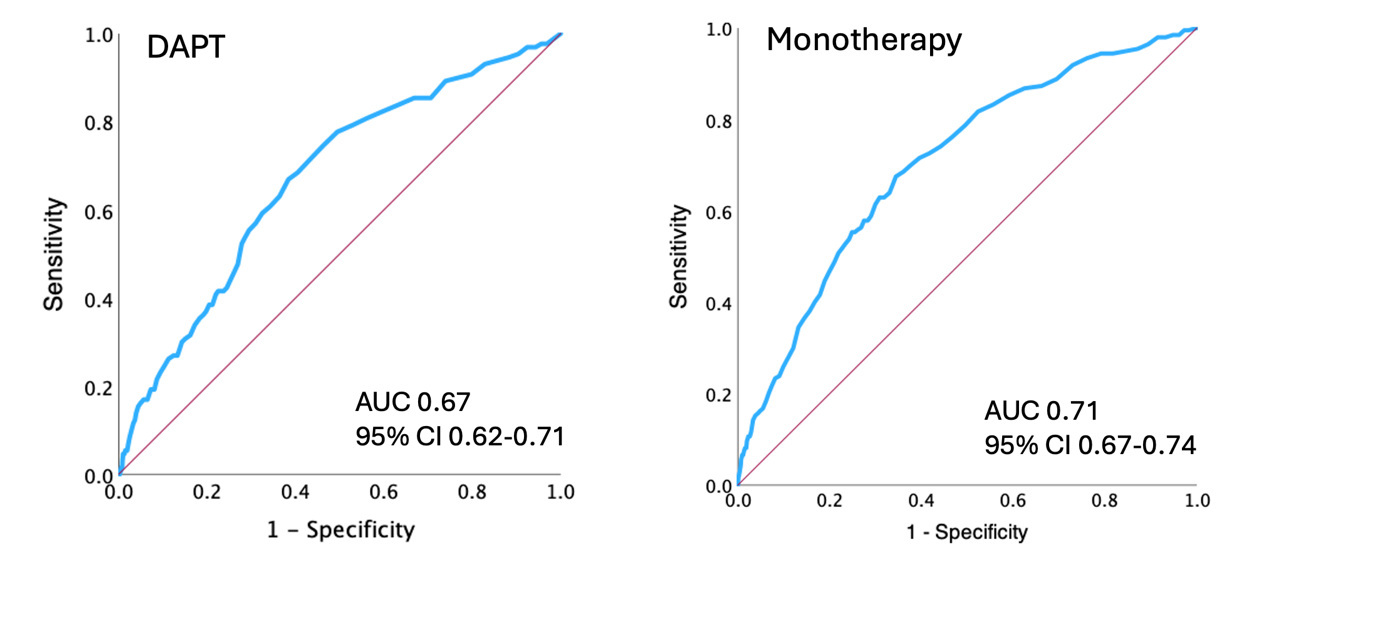

Supplement: pvae060_Supplemental_File [file pvae060_supplemental_file.docx]
